# Development of High-Performance Hot-Deformed Neodymium–Iron–Boron Magnets without Heavy Rare-Earth Elements

**DOI:** 10.3390/ma16196581

**Published:** 2023-10-06

**Authors:** Keiko Hioki

**Affiliations:** Corporate Research & Development Center, Daido Steel Co., Ltd., 2-30 Daido-cho, Minami-ku, Nagoya 457-8545, Japan; k-hioki@ac.daido.co.jp

**Keywords:** hot-deformed Nd-Fe-B magnet, heavy rare-earth element, coercivity, microstructure

## Abstract

Neodymium–iron–boron magnet is an essential material for the traction motors of green vehicles because it exhibits the highest maximum energy product, (*BH*)_max_, out of all permanent-magnet materials. However, heavy rare-earth elements such as dysprosium and terbium, which are scarce resources, are added to these magnets to improve their heat resistance. To address this resource problem, considerable efforts have been made to reduce the composition of heavy rare-earth elements in these magnets without causing a significant reduction in coercivity. Hot-deformed Nd-Fe-B magnets are a category of Nd-Fe-B magnets where precious materials such as heavy rare-earth elements can be eliminated or reduced to maintain high coercivity owing to their fine microstructure. Although they are not often used for the fabrication of high-performance magnets due to their complicated production process and the difficulty in controlling their fine microstructure, after the rare-earth crisis in 2020, these magnets have begun to attract attention as a material that could increase coercivity when controlling their microstructures. This paper provides an overview of hot-deformed magnets and the efforts made to improve their properties by controlling their microstructures.

## 1. Introduction

**Figure 1:**
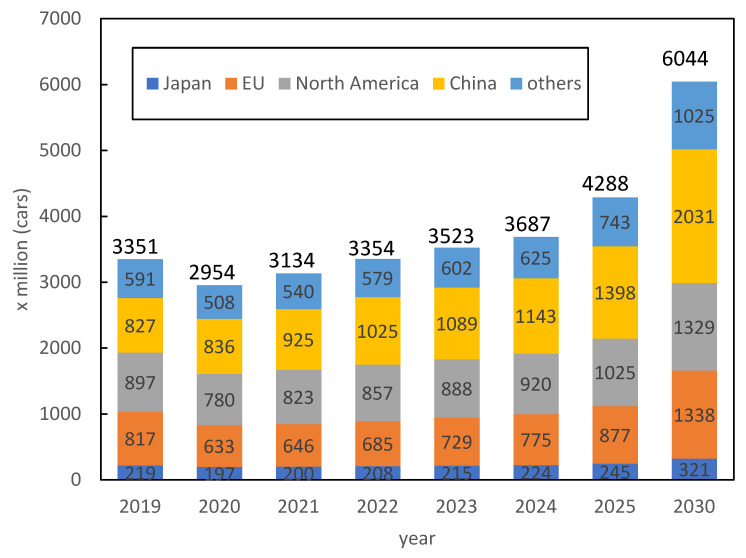
Market size forecast for automotive motors [1].

**Figure 2:**
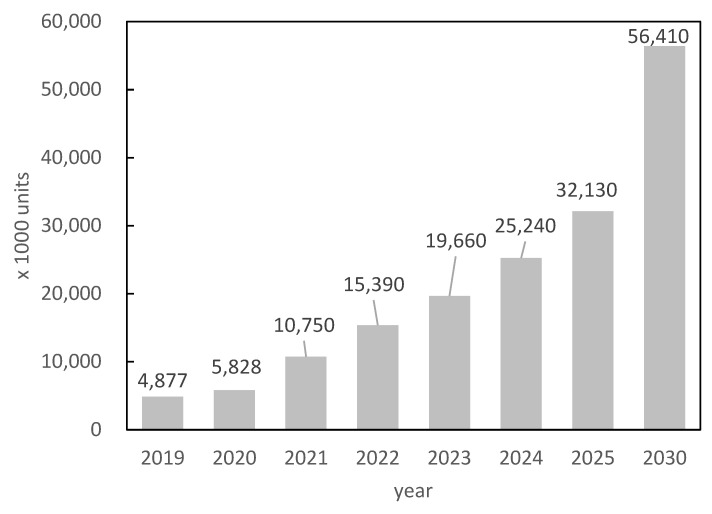
Market size forecast for traction motors based on number of vehicles [1].

In recent years, the development of eco-friendly vehicles has been promoted to address environmental issues. Figure 1 shows the sale quantity for vehicle motors, which is expected to reach up to 145% in 2025 and 205% in 2030 compared to 2020 [1]. The growth of the traction motor market is particularly expected to exceed 10 times by 2030, as shown in Figure 2 [1]. Neodymium–iron–boron magnets [2], with the highest known (*BH*)_max_ values, are generally used in permanent-magnet synchronous motors, which are the mainstream traction motors. However, these magnets have a relatively low Curie temperature and show significant coercivity degradation at high temperatures, considering the motor working environment. Therefore, heavy rare-earth elements (HREEs), such as Dy and Tb, have conventionally been replaced (up to approximately 10 wt.%) with light rare-earth elements such as Nd and Pr to increase the anisotropic magnetic field of the crystal and improve coercivity [3]. Although this method is a facile way to increase coercivity, *H*_cj_, in industrial production, it also causes degradation of saturation magnetization, *J*_s_. Therefore, the development of technology to reduce HREE consumption without degrading coercivity is an important and challenging issue in the magnet industry; techniques such as grain boundary diffusion [4,5] and microstructure engineering (reducing grain size [6] and modifying the grain boundary phase [7,8,9]) have been proposed to address this issue.

Nd-Fe-B sintered magnets are widely used as high-performance magnets owing to their high industrial productivity and possession of the highest maximum energy product, (*BH*)_max_, among all permanent magnet materials [2]. On the other hand, the rapidly quenched Nd-Fe-B powder invented by Croat et al. was initially only considered suitable for isotropic bonded magnets as it consists of a polycrystalline structure with randomly oriented crystals of several tens of nanometers in size [10]. This structure cannot be crushed into single crystals, and orienting the *c*-axis of the grains in the same direction by applying an external magnetic field is not possible. Nevertheless, hot-deformed magnets were introduced, where the crystals are mechanically oriented via hot plastic deformation (thermomechanical alignment), resulting in a magnet with crystal grains approximately 1/10 finer than those in an ordinarily sintered Nd-Fe-B magnet [11]. Due to this microstructural control, hot-deformed magnets are regarded as having the potential to reduce the consumption of HREEs, especially since the rare-earth crisis of 2020. However, it was also pointed out that their coercivities are not as high as expected from their grain size [12], and their remanences, *B*_r_, are slightly lower than those of sintered magnets. This is likely because their production requires a high-level melt-spinning process to prepare high-quality raw powders. Moreover, a complicated production process is necessary to obtain a high-density and highly oriented anisotropic magnet.

In this article, we provide basic information about hot-deformed magnets, describe their production process in Section 2, and present their features in Section 3 before discussing recent development studies in detail in Section 4. We hope this work will provide useful information to readers, especially manufacturers of permanent magnets.

## 2. Production Process of Hot-Deformed Nd-Fe-B Magnets

### 2.1. Production Process

**Figure 3:**
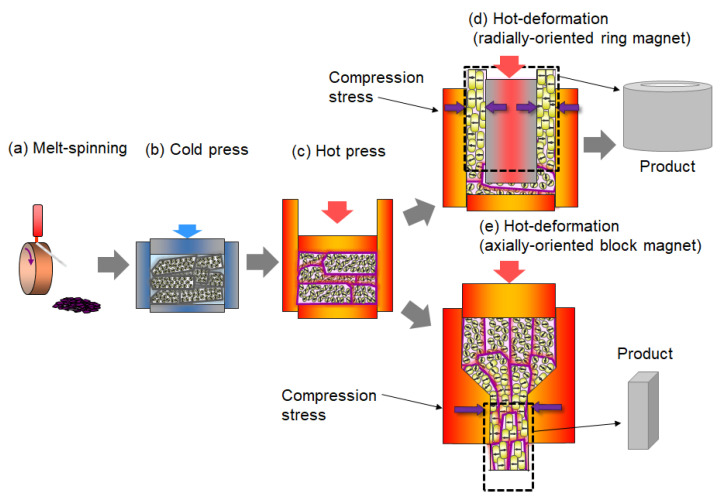
Production process of radially oriented hot-deformed ring magnet and axially oriented plate magnet.

The entire manufacturing process of hot-deformed magnets consists of two steps: raw material production via melt spinning and fabrication of a full-dense anisotropic magnet using the melt-spinning powder as the initial raw material. Figure 3 presents a schematic of the production process for a radially oriented ring magnet (backward extrusion) and an axially oriented plate magnet (forward extrusion). The lateral profiles of both magnets are shown in Figure 3d,e, respectively.

The thickness of ribbons produced via melt spinning is 20–30 μm (Figure 3a). One of the challenges of the melt-spinning technique is producing raw materials with a uniform microstructure on a mass production scale. Even within a ribbon thickness range of 20–30 μm, the cooling rate varies from the roller surface to the free surface, resulting in ribbons with a grain-size distribution in the thickness direction. This grain-size distribution in the ribbon directly impacts the microstructure’s uniformity in the final product. Therefore, raw material manufacturers have made significant efforts to provide raw materials with uniform microstructures.

Thin ribbons produced via the melt-spinning method are pulverized into flake powder with a diameter of approximately 150 μm as the initial raw material (Figure 3a). This raw powder is a nano-polycrystalline material consisting of numerous randomly oriented Nd_2_Fe_14_B crystal grains with a diameter of approximately 10–30 nm. The chemical composition of the powder is slightly richer in rare-earth elements when compared to the stoichiometry of the Nd_2_Fe_14_B phase to supply a liquid phase during the hot-deformation process at high temperatures, which becomes the grain boundary phase surrounding the Nd_2_Fe_14_B grains in the final product. The raw powder is cold pressed at room temperature and hot pressed at approximately 800 °C to obtain a fully dense body (Figure 3c). The resulting hot-pressed body is an isotropic magnet with grains that are approximately 20–50 nm in size. The hot-pressed body is then hot deformed at approximately 800 °C (Figure 3d,e) to obtain an anisotropic magnet. This is the point at which the thermo-mechanical alignment of the crystal grains occurs, resulting in a larger grain size and aspect ratio (diameter of 200–500 nm and thickness of 20–50 nm). During hot deformation, the *c*-axis of Nd_2_Fe_14_B crystals is gradually oriented to be parallel to the compression direction. The hot-pressing and hot-deformation temperatures are kept higher than the melting point of the grain boundary phase to ensure good formability during pressing. However, excessive heat input during hot deforming is one of the main reasons for abnormal grain growth in the final product [13,14]. Thus, optimizing the mold design and hot-deformation conditions is important for this kind of magnet.

### 2.2. Crystal Orientation Mechanism

Many microstructural analyses using transmission electron microscopy (TEM) have been performed to clarify the orientation mechanism of this kind of magnet since Lee invented a die-upset anisotropic magnet [11]. Although changes in the microstructure during die upsetting were observed, and several crystal orientation mechanisms were proposed, they have not been completely confirmed. In this section, we present our research results [15] and possible mechanisms based on previous studies.

**Figure 4:**
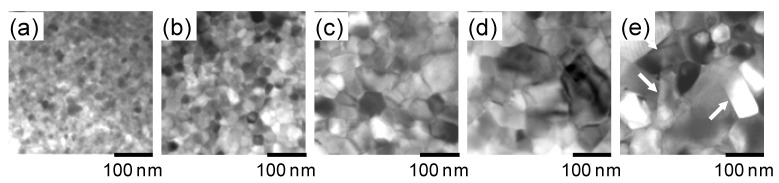
TEM images of rapidly quenched ribbons annealed at 750 °C for (**a**) 0 min (as quenched), (**b**) 1 min, (**c**) 3 min, (**d**) 5 min, and (**e**) 10 min (platelet grains are indicated by arrows) [15].

**Figure 5:**
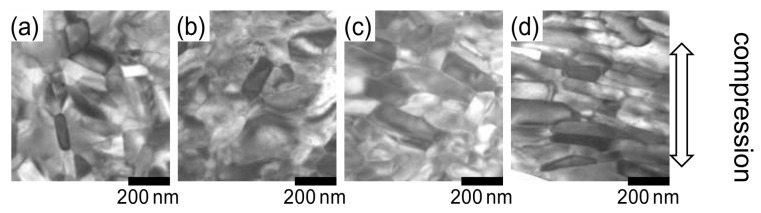
TEM images of die-upset magnets (perpendicular to the compression force direction) with a compression ratio, *R*, of (**a**) 0%, (**b**) 20%, (**c**) 40%, and (**d**) 60% [15].

The TEM images of raw powders with a typical composition before and after heat treatment at 750 °C for different durations (0 min (as quenched), 1 min, 3 min, 5 min, and 10 min) are shown in Figure 4a–e, whereas Figure 5a–d present the TEM images of die-upset samples at 850 °C with various reduction ratios. The TEM images in Figure 5 are taken along the compression direction, which is the vertical direction in the images. The reduction ratio, *R*, is calculated using Equation (1) [15], where *h*_0_ and *h* are the sample height before and after die upsetting:

*R* = (*h*_0_ − *h*)/*h*_0_ × 100 (1)

After analyzing Figure 4, an increase in heat-treatment time is considered to have led to some grains in the raw powder growing anisotropically without alignment (indicated by arrows in Figure 4e), even without compression stress. A previous study showed that the preferential grain growth direction is perpendicular to the *c*-axis of tetragonal Nd_2_Fe_14_B crystals [16]. As evident in Figure 5, both grain size and aspect ratio increase gradually, and grain alignment progresses as the compression ratio increases. No dislocations or slip lines were observed in the microstructure during these changes, indicating that the microstructure change was not caused by the mechanisms of dislocations [14].

**Figure 6:**
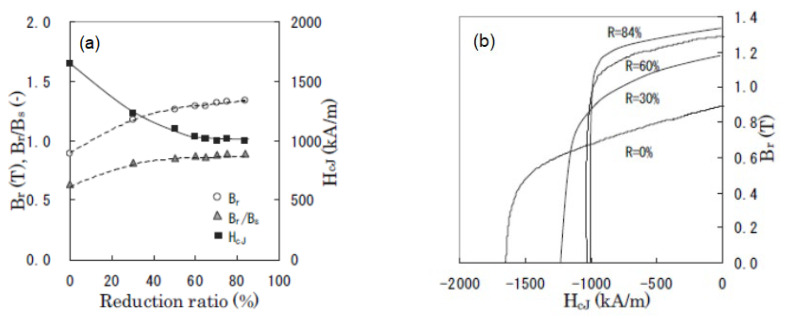
(**a**) Variations in the reduction ratio of magnetic properties (*B*_r_ and *H*_cJ_) and alignment (*B*_r_/*B*_s_). (**b**) Demagnetization curves for hot-pressed samples (reduction ratio is 0%) and die-upset samples (reduction ratios are 30, 60, and 84%). Upsetting temperature is fixed at 780 °C, and strain rate is 3.33 × 10^−2^ s^−1^ [17].

Figure 6a illustrates the correlation between magnetic properties (*B*_r_ and *H*_cj_) and alignment (*B*_r_/*B*_s_) and the reduction ratio of hot-deformed samples at constant temperatures and strain rates, while Figure 6b displays the corresponding demagnetization curves [17]. These properties were measured using a pulsed high-field magnetometer. Here, we define the magnetization at *H* = 5420 kA/m as *B*_s_, which is the saturation magnetization. The chemical compositions of the samples shown in Figure 4, Figure 5 and Figure 6 are nearly identical. It is evident that as the compression ratio increases, *B*_r_ also increases due to increasing grain orientation, and *H*_cj_ decreases due to grain growth.

**Figure 7:**
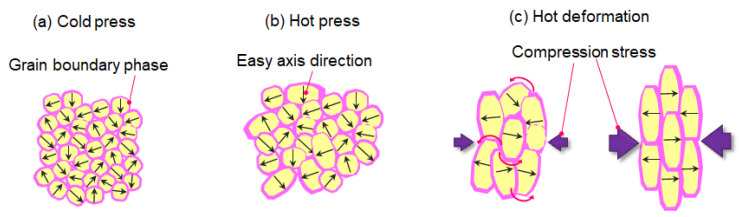
Schematic models of change in microstructure for (**a**) cold-pressed body, (**b**) hot-pressed body, and (**c**) hot-deformed magnet.

Therefore, based on previous studies, whose results are described in Figure 4, Figure 5 and Figure 6, it can be inferred that during the hot-deforming process, the grain growth (particularly anisotropic growth) and the melting of the grain boundary phase occur at the hot-deforming temperature. Subsequently, preferential grain growth progresses, and crystal rotation occurs with grain boundary sliding on the c-plane, which is the larger plane of platelet-shaped grains, predominantly under heat and compression stress (Figure 7a,b). In this process, the liquid phase plays a role as a lubricant. Finally, the grain shape changes into a platelet, and the *c*-axis of the grain orients is parallel to the compression stress direction (Figure 7c). Additionally, it was reported that the chemical composition in the grain boundary phase changes along with grain growth during the hot-deformation process [18]. Thus, it is inferred that atom transfer via the grain boundary phase promotes preferential grain growth under compression stress. However, the principle of preferential grain growth under compression stress has not been fully elucidated, even though some research studies have proposed theories such as “grain boundary migration” [13,14,19] and “interface-controlled solution-precipitation creep” [20,21]. The latter is promoted under high stress: atoms on the surface are absorbed in the grain boundary phase under higher stress (c-plane) and precipitate on the surface under less stress (ab-plane). In any case, hot deformation is usually completed in several tens of seconds, meaning that significant microstructural changes are completed in a short time because grain growth and orientation are synergistic changes.

### 2.3. Efforts for Manufacturing Commercial Products

Initially, disk-shaped die-upsetting magnets were only fabricated for basic research purposes. In 1988, Kojima et al. reported the first ring-shaped hot-deformed magnet designed to be mounted on motors [22]. They used backward extrusion to fabricate the ring-shaped magnet and forward extrusion to fabricate the column-shaped magnet. They found that the ring-shaped magnet fabricated via backward extrusion exhibited a strong radial orientation. Yoshida et al. conducted further investigations on compression stress and grain alignment during the hot-deformation process [23]. They fabricated four different types of hot-deformed magnets (disk-shaped die-upsetting magnet, forward-extruded ring-shaped magnet, backward-extruded ring-shaped magnet, and forward-extruded column-shaped magnet) with a wide range of compression ratios. Their basic research also showed that the radially oriented ring-shaped hot-deformed magnet, which is suitable as a motor material, can be produced via backward extrusion.

In 1992, Daido Electronics began commercializing radially oriented ring-shaped hot-deformed magnets based on the aforementioned results [23]. Ring magnets fabricated using backward extrusion during hot deformation have crystal grains that are uniformly oriented in the radial direction without applying a magnetic field. As a result, it is easier to obtain ring magnets with a small diameter or great height, which are typically difficult to fabricate via sintering. Furthermore, magnets fabricated using this method do not have an orientation distribution in the ring length direction and possess a fine microstructure, thereby achieving a smooth magnetization pattern.

**Figure 8:**
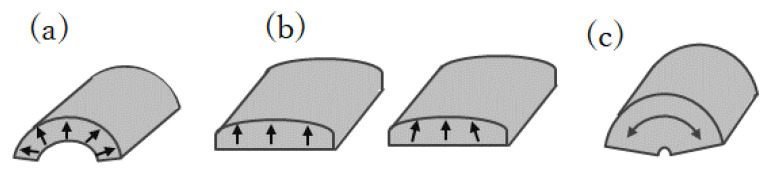
Shapes and orientation of hot-deformed magnets: (**a**) arc-shaped magnets with radial orientation, (**b**) bread loaf-shaped magnets with radial and axial orientations, and (**c**) fan-shaped magnets with circumferential orientation.

Plate-shaped magnets have traditionally been used for mounting on the traction motors of HEVs. To accommodate hot-deformed magnets in an HEV’s motor design, plate-shaped hot-deformed magnets, as shown in Figure 3, were commercialized in 2016 by Daido Electronics. Additionally, Figure 8 illustrates further development of flexible shape extrusion, which has resulted in the creation of (a) arc-shaped magnets with radial orientation, (b) bread-loaf-shaped magnets with radial and axial orientations, and (c) fan-shaped magnets with circumferential orientation. These new shapes of hot-deformed magnets were designed to cater to the requirements of modern motor designers.

## 3. Features of Hot-Deformed Magnets

### 3.1. Microstructure

**Figure 9:**
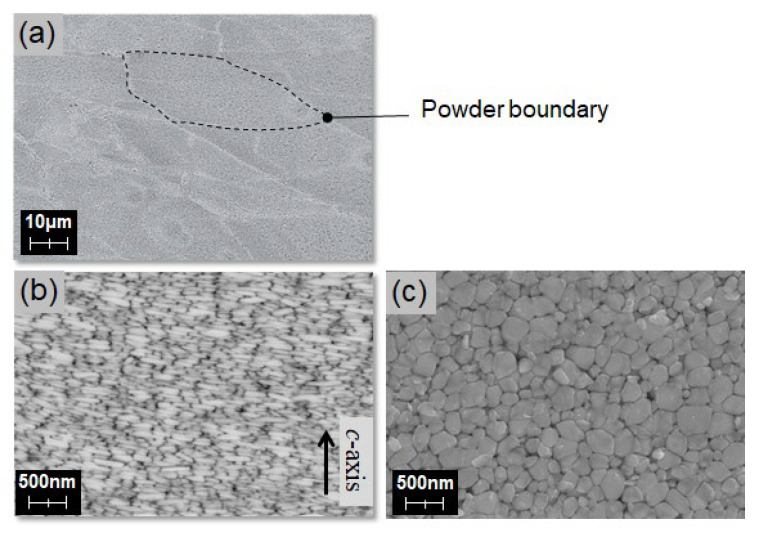
SEM images for (**a**) typical hot-deformed magnet [24] observed from the plane (**b**) parallel [24] and (**c**) perpendicular to the *c*-axis.

The SEM images of a typical hot-deformed magnet are shown in Figure 9a,b [24], and Figure 9c. The observation planes were mirror polished, followed by etching. The dashed line in Figure 9a shows the powder boundary of the original raw powder, which is defined from its optical microstructure images that are observed separately, whereas Figure 9b,c show the SEM images of the planes perpendicular and parallel to the c-axis, respectively. These images reveal that the hot-deformed magnet has a structure in which flat grains are stacked in the *c*-axis direction. The grains are about one order of magnitude finer than those of a typical sintered magnet, which has approximately the same critical single-domain grain size (0.3 μm) of a Nd-Fe-B magnet [25]. Grain size refinement was also performed for sintered magnets on a laboratory scale, reaching a diameter of 1 μm [6], and on a mass production scale, reaching 3 μm. However, it is difficult to avoid oxidation when dealing with the raw material of sintered magnets, which contains single crystals that are finer than the raw powder obtained after melt spinning. Therefore, a fine microstructure remains the most significant feature of hot-deformed magnets.

### 3.2. Magnetization Curve and Magnetic Domain Pattern

**Figure 10:**
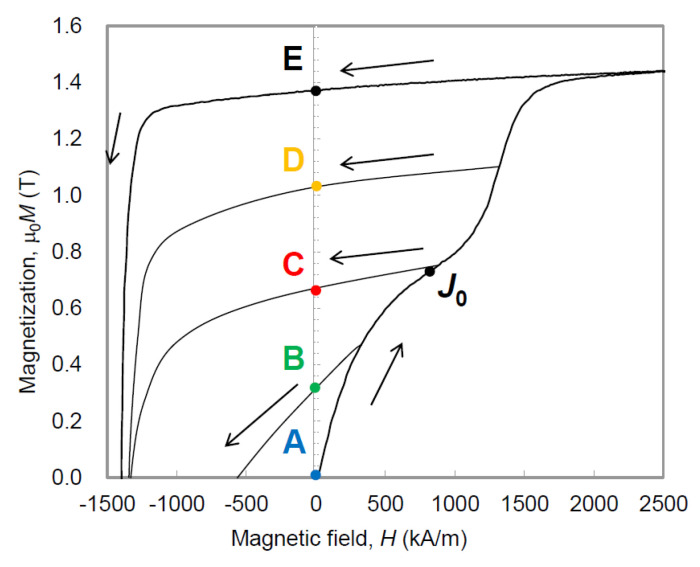
Initial magnetization and demagnetization curves for sample C. *J*_0_ is an inflection point of the initial magnetization curve. The magnetization curves indicated as B–E were measured using a pulsed high-field magnetometer with applied magnetic fields *H* = 279.2, 893.4, 1292, and 5991 kA/m (3.5, 11.2, 16.2, and 75.1 kOe), respectively. Point A shows the thermally demagnetized state [26].

Figure 10 shows the initial magnetization and demagnetization curves of a hot-deformed magnet with the composition of Nd_13.5_(Fe, Co)_80.29_B_5.64_Ga_0.57_ (at.%) at RT; the detailed sample preparation process is provided in ref. [26]. Magnetization curves with applied magnetic fields *H* = 279.2, 893.4, 1292, and 5991 kA/m (3.5, 11.2, 16.2, 75.1 kOe) were measured using a pulsed high-field magnetometer. Point A shows the thermally demagnetized state. It is evident that the initial magnetization curve exhibits two distinct steps with two inflection points, indicating that this magnet comprises two different magnetic phases. Generally, the first and second curves are interpreted as nucleation-type and pinning-type areas consisting of multi-domain grains and single-domain grains, respectively.

**Figure 11:**
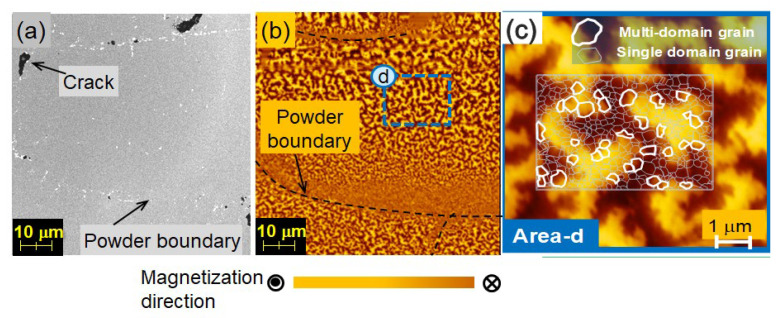
(**a**) SEM and (**b**) MFM images of a typical hot-deformed magnet (c-plane), with (**a**) and (**b**) showing the same area, while (**c**) is an MFM image showing area d. Contours of grains obtained from the SEM images are overlaid on the MFM images [26].

Figure 11a,b depict the same region viewed under an optical microscope and a magnetic force microscope (MFM) in the thermally demagnetized state, respectively, and the plane of observation is the c-plane. In Figure 11c, an image of the magnetic domain of area d in Figure 11b is shown, which is also observed using MFM. The white lines represent the contours of the crystal grains, which are observed separately using LV-SEM. The bright and dark shaded areas represent the two magnetic polarizations, N and S, respectively [26].

In the thermally demagnetized state, the magnetic domain structure of the hot-deformed magnet exhibits a maze pattern, as shown in Figure 11b,c. It is observed that the magnetic domains consist of groups of single-domain grains, although multi-domain grains are present at the edges of the maze pattern (indicated by the thick white line). Magnetic walls in the areas where single-domain grains exist are predominantly located along the grain boundary phase, suggesting the presence of pinning at the grain boundary phase. This behavior can be attributed to the existence of magnetic walls along the grain boundaries, which serve to reduce the magnetic wall energy.

**Figure 12:**
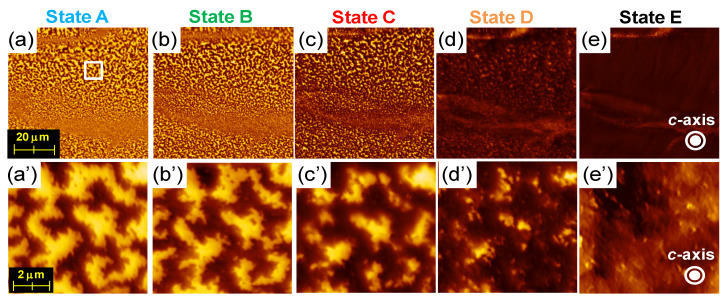
(**a**–**e**) Magnetic domain structures under *H* = 0 after applying the magnetic fields *H* = 3.5, 11.2, 16.2, and 75.1 kOe, corresponding to A–E. (**a′**–**e′**) are the enlarged figures of the area surrounded by the white square in (**a**). (**a**–e) and (**a′**–**e′**) are observed in the same areas [26].

Figure 12 shows the MFM images of the same areas after applying a specific magnetic field, followed by returning to *H* = 0, corresponding to states A–E in Figure 10. As the magnetic field is applied, the width of the magnetic domains expands continuously and appears to spread mainly via propagation.

Okamoto et al. conducted a detailed investigation of the changes in the magnetic domain structure during the initial magnetization and demagnetization processes using X-ray magnetic circular dichroism (XMCD) and LV-SEM [27]. They reported a completely new observation: the magnetic walls move across the multi-domain grains under the magnetic fields. The multi-domain grains always exist at the edge of the maze pattern during the magnetization process, although their volume (number) decreases as the magnet is fully magnetized. They also found that such areas, which are difficult to fully magnetize, are also difficult to demagnetize; the magnetic domains exist until the applied magnetic field is close to its coercivity. It can be presumed that such areas serve as strong pinning areas for the magnetic walls; however, no special features, such as grain size and grain shape, are observed in the microstructure.

## 4. Recent Developments in Hot-Deformed Magnets

### 4.1. Effects of Microstructure and Composition on Magnetic Properties

The magnetic properties of hot-deformed magnets are strongly influenced by their chemical compositions and microstructures. Therefore, in a previous study, we conducted a systematic evaluation of the effects of chemical composition and microstructure such as grain size, composition, and thickness of the grain boundary phase on the magnetic properties of hot-deformed Nd-Fe-B magnets [28].

To conduct this investigation, five types of samples with different chemical compositions (A–E) were prepared and hot deformed at various temperatures, as shown in Table 1. Samples A–C and D and E were specifically prepared to examine the effects of grain size, grain boundary phase, and the effects of Dy addition, respectively. The details of the sample preparation process are provided in the referenced source.

**Figure 13:**
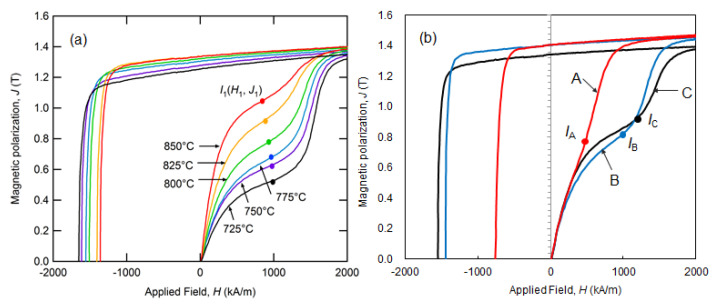
Initial magnetization and demagnetization curves for (**a**) sample C following deformation at 725, 750, 775, 800, 825, and 850 °C [28]. (**b**) Samples A, B, and C hot deformed at 775 °C.

Figure 13a,b show the initial magnetization and demagnetization curves of sample C hot deformed at various temperatures and samples A–C hot deformed at 775 °C, respectively. These magnetization curves with an applied magnetic field *H* = 5991 kA/m (75.1 kOe) were obtained using a pulsed high-field magnetometer. As shown in Figure 13a, an increase in hot-deformation temperature leads to a higher *B*_r_ while *H*_cj_ becomes lower. Other samples show the same trend. Moreover, it is observed that the ratio of the first step in the initial magnetization curves (*I*) also increases continuously as the hot-deformation temperature increases.

In Equation (2) [28], the volume ratio of single-domain grains, SDGR, is defined based on the initial magnetization curve, where *J*_1_ is the inflection point in Figure 13 and *J*_s_ is the saturation magnetization.

SDGR = 1 − *J*_1_/*J*_s_ (2)

**Figure 14:**
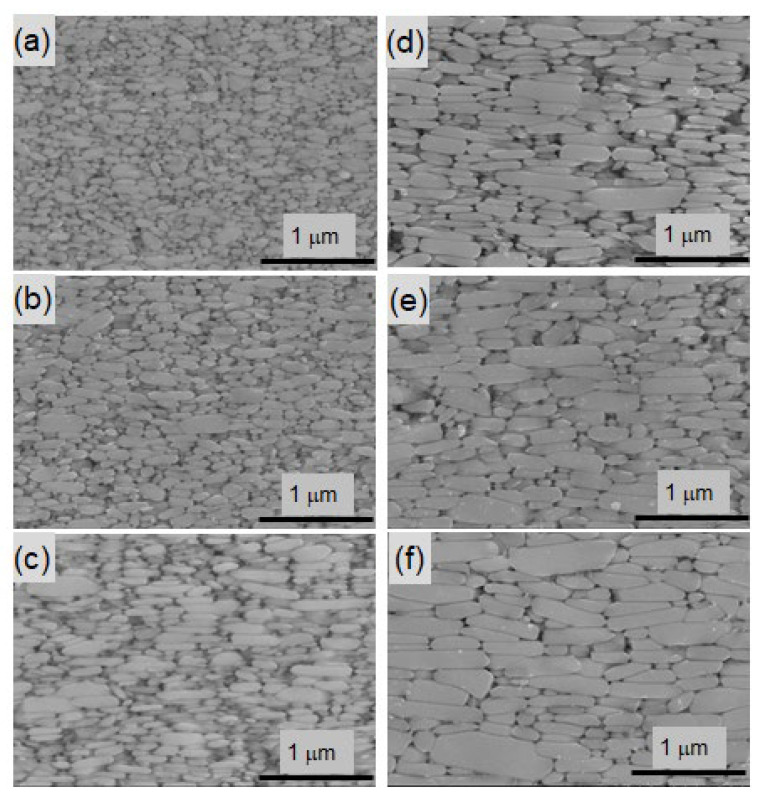
SEM images parallel to the *c*-axis for sample C after deformation at (**a**) 750 °C, (**b**) 775 °C, (**c**) 800 °C, (**d**) 825 °C, (**e**) 850 °C, and (**f**) 875 °C [28].

Figure 13b displays the magnetization curves for samples A–C with different Nd contents. It can be observed that the inflection points shift toward higher magnetic fields as the Nd concentration increases, which is attributed to the microstructure (grain boundary phase). Additionally, Figure 14a–f show the SEM images of sample C hot deformed at 725–850 °C, where the observation planes are parallel to the *c*-axis. The average grain size is defined as the diameter in the longitudinal direction of the platelet grains of such SEM images.

**Figure 15:**
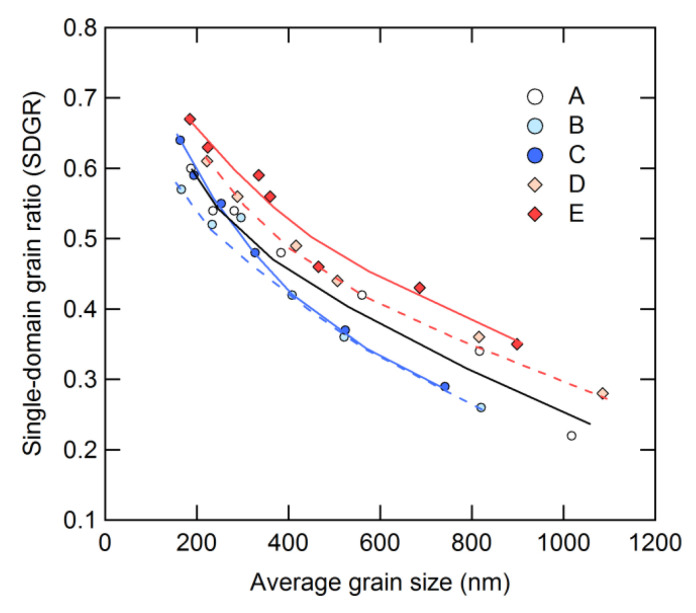
Dependence of the single-domain grain size (SDGR) on the average grain size for samples A–E. The lines are visual aids [28].

The relationship between SDGR, calculated based on Equation (2), and the average grain size defined from the SEM images is presented in Figure 15 [28]. The results show that SDGR decreases with an increase in average grain size for all compositions. As can be seen from Figure 14 and Figure 15, the grains grow as the hot-deformation temperature increases, indicating that the initial magnetization curves provide insights into the microstructure.

**Figure 16:**
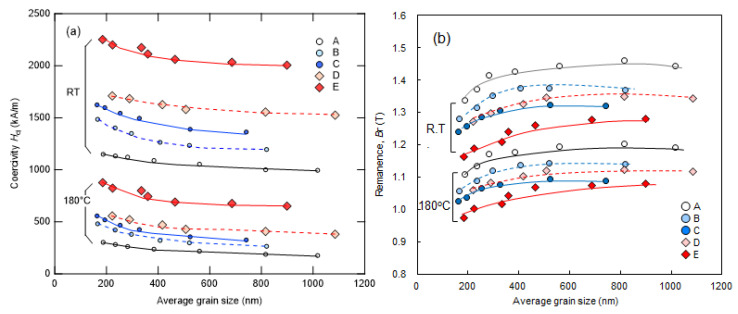
Dependence of (**a**) coercivity [28] and (**b**) remanence on the average grain size for samples A–E at room temperature and 180 °C. The lines provide visual guides.

Figure 16a,b depict the relationship between the average grain size defined from the SEM images and *H*_cj_ and *B*_r_ at RT and 180 °C obtained using a BH tracer, respectively. As shown in Figure 16a, the coercivity of all samples A-E increases as the average grain size decreases. Notably, coercivity can be increased not only by adding Dy but also by increasing the total amount of rare-earth elements (TREs). Additionally, at 180 °C, the coercivity of composition C with an average grain size of 400 nm or less (Dy = 0) is comparable to composition D with an average grain size of 800 nm (Dy = 1.0 at.%). In Figure 16b, it is observed that remanence decreases with decreasing average grain size because grain alignment requires a certain degree of preferential grain growth, as explained in Section 2.2. However, excess heat input promotes abnormal grain growth, subsequently hindering neighboring grains from aligning and resulting in degradation of remanence.

**Figure 17:**
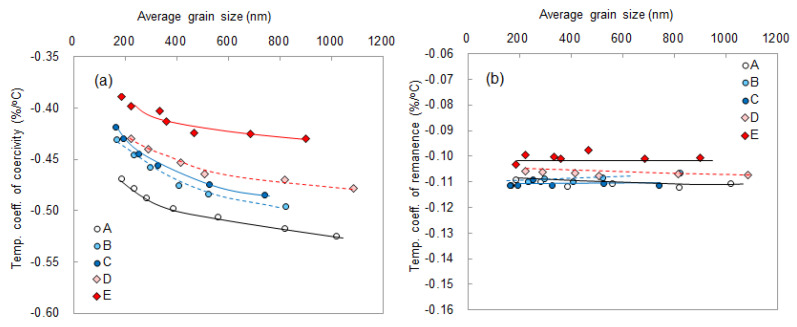
Dependence of (**a**) temperature coefficient of coercivity and (**b**) temperature coefficient of remanence on the average grain size for Samples A–E. The lines provide visual guides [28].

Figure 17a,b show the dependence of temperature coefficients of coercivity and remanence on the average grain size, respectively. Here, α and *β* are determined following [29]. The coercivity and remanence at RT and 180 °C, as calculated using Equations (3) and (4) are referred from Figure 16:

*β* = (*H*_cj_ (23 °C) − *H*_cj_ (180 °C))/((23 °C − 180 °C)) × 100/(*H*_cj_ (23 °C)) (3)

*α* = (*B*_r_ (23 °C) − *B*_r_ (180 °C))/((23 °C − 180 °C)) × 100/(*B*_r_ (23 °C)) (4)

From Figure 17b, it can be observed that the temperature coefficient of remanence, *α*, improves slightly with the addition of Dy and is independent of the total rare-earth element content and grain size. Conversely, the temperature coefficient of coercivity, *β*, is improved not only by adding Dy but also by increasing TRE and decreasing the grain size. This result suggests that it is possible to obtain a temperature coefficient of coercivity, *β*, comparable to that obtained via Dy addition via grain size refinement. Similar results were also observed in sintered magnets [29].

In a study conducted by Liu et al., microstructure analysis was performed on HREE-free hot-deformed magnets with different TRE amounts to investigate the relationship between grain boundary conditions and coercivity [30]. The samples they observed had Nd contents of 12.7, 13.0, and 14.0 at.%, showing similar compositions to samples A–C in Table 1. The coercivities of these samples were 720 kA/m (9.0 kOe), 1030 kA/m (12.9 kOe), and 1430 kA/m (17.9 kOe), respectively, with coercivity increasing as the total rare-earth element content increased. The samples were observed using TEM, LV-SEM, and 3DAP (three-dimensional atomic probe). The thickness of the grain boundary phase was found to increase in the order from A to C; the average Nd concentration in the grain boundary phase also increased from 22.7 at.% to 46.0 at.% in the order from A to C. These results suggest that an increase in the total rare-earth element content of the magnet improves the isolation among grains magnetically, thereby leading to higher coercivity.

Another microstructural analysis of the effect of grain size was also reported in [31]. Sepheri-Amin et al. performed microstructural analysis on samples with Nd = 13.0 at.% (similar to sample B in Table 1), which were hot deformed at various temperatures. The results show that coercivity decreases as the hot-deformation temperature increases due to an increase in grain size. The microstructure analysis revealed that hot deformed samples at lower temperatures exhibited fine and uniform microstructures, with high Nd contents in the grain boundary phases. In contrast, samples that were hot deformed at higher temperatures have abnormally coarse grains but large triple junctions, resulting in thin normal grain boundary phases with relatively lower Nd contents. This analysis suggests that the coercivity and heat resistance of hot-deformed magnets can be improved via hot deforming at low temperatures due to both grain size refinement and the isolation among grains magnetically.

These aforementioned studies found improvements to the heat resistance of hot-deformed magnets via the control of the microstructure without the addition of HREEs.

### 4.2. Improving Uniformity of Microstructure

**Figure 18:**
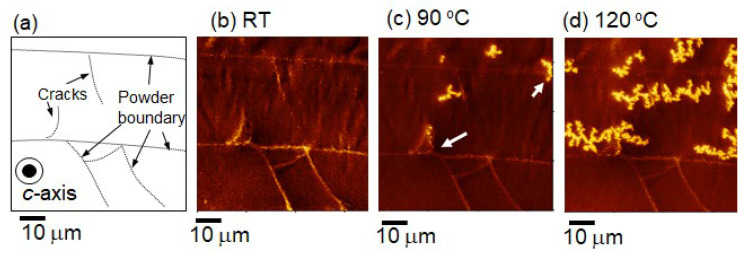
Images of (**a**) contours and cracks of powders obtained using SEM, and (**b**–**d**) magnetic domain pattern during the thermal demagnetization process observed using MFM at RT, 90, and 120 °C.

**Figure 19:**
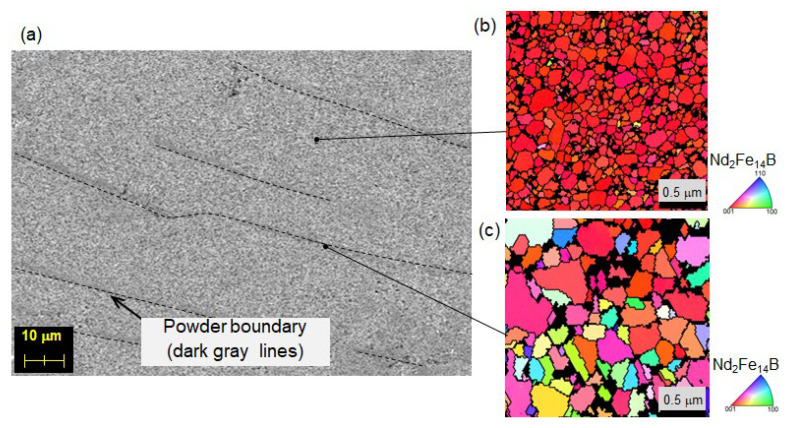
(**a**) SEM and (**b**,**c**) EBSD images of typical hot-deformed magnet (c-plane).

**Figure 20:**
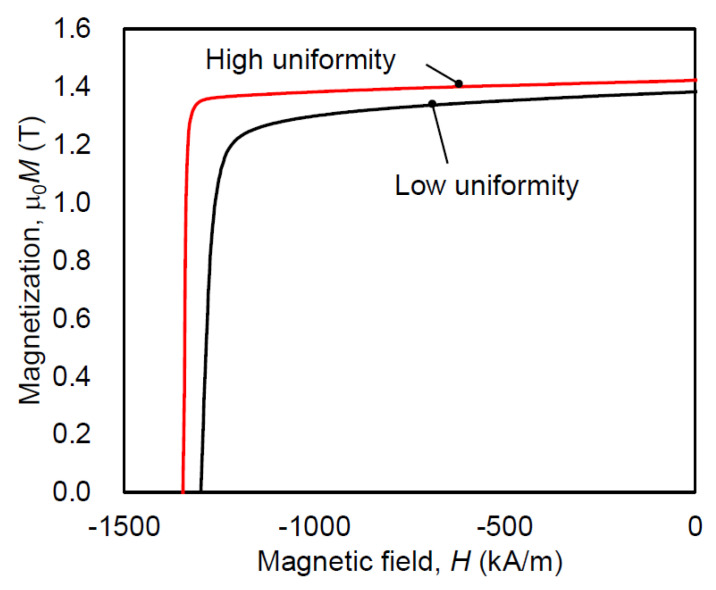
Demagnetization curves of hot-deformed magnets before and after the reduction in coarse and misaligned grains at the powder boundaries.

To determine the starting point of demagnetization, the changes in the magnetic domain structure during thermal demagnetization were observed using MFM. The SEM image of the contours and cracks of the powders of the same sample, as described in Section 4.1, is taken perpendicular to the *c*-axis and shown in Figure 18a, whereas Figure 18b–d show the corresponding magnetic domain structures in the same area at room temperature, 90 °C and 120 °C, respectively. The experiment involved in situ observation of the magnetic domain structure during the thermal demagnetization process of a fully magnetized sample at a given temperature. The magnetic domain image at RT reveals that almost all crystal grains are magnetized in the same direction. However, the image at 90 °C shows some magnetization reversals starting at the powder boundary, as indicated by the arrows. By contrast, the optical microscope image shows that magnetization reversal tends to occur from coarse, equiaxed grains that exist at some powder boundaries (Figure 19). To reduce these areas, it is crucial to increase the uniformity of the raw powder’s microstructure, as described in Section 2.1, and to avoid excessive heat input by optimizing the mold design and hot-deformation conditions. After improving the microstructure’s homogeneity, the demagnetization curves, measured using a BH tracer and shown in Figure 20, indicate that coercivity increases due to a reduction in coarse grains and remanence improves as the number of equiaxed grains decreases.

## 5. Conclusions and Future Developments

The findings presented in Section 4.1 and Section 4.2 demonstrate the potential of controlling the microstructure of hot-deformed Nd-Fe-B magnets, including grain size, grain boundary phase thickness, and composition, on a submicron scale by optimizing the hot-deformation conditions and chemical compositions, particularly the rare-earth element content. Notably, the manufactured HREE-free hot-deformed magnet based on these basic research results achieves coercivity exceeding 1600 kA/m (20 kOe), and it was used for traction motors of HEVs for the first time in the world [32]. In addition to the findings presented above, other promising approaches to improve the performance of permanent magnet as a motor material and address resource issues include the following:

(1) The development of hot-deformation techniques to produce various magnet shapes with different grain orientations, as described in Section 2.3, to provide magnets for newly designed motors [33].
(2) The application of a grain boundary modification technique for hot-deformed Nd-Fe-B magnets using a small amount of heavy rare-earth alloy or heavy rare-earth free alloy to dramatically improve coercivity [34,35,36].
(3) The use of abundant and low-cost rare-earth elements, such as Ce and La, in a well-balanced manner to reduce the use of not only HREEs but also Nd and Pr. Although the physical properties of R_2_Fe_14_B phases with R=Ce are lower than those of R=Nd, Pr, the use of Ce was shown to improve formability and reduce grain growth during hot deformation, resulting in a finer microstructure [37,38,39].
(4) Increasing the electrical resistivity of magnet materials is necessary. During motor operation, the temperature on the surface of a magnet increases due to eddy currents. One effective method to reduce this is increasing the electrical resistivity of magnet materials. Studies have shown that mixing high-electrical-resistivity materials with raw powders, followed by magnet fabrication, can achieve this increase for hot-deformed magnets [40,41].

## Figures and Tables

**Table 1:** Chemical compositions and hot-deformation temperatures of the samples [28]. Bal. (Balance) means the leftover percentage.

Sample	Chemical Composition (at.%)						Hot-Deformation Temperature
	Nd	Dy	Fe	Co	B	Ga	(°C)
A	12.8	0	Bal.	3.85	5.65	0.46	750, 775, 800, 825, 850, 825, 850
B	13.5	0	Bal.	3.82	5.64	0.57	750, 775, 800, 825, 850, 875
C	14.2	0	Bal.	3.81	5.66	0.71	725, 750, 775, 800, 825, 850
D	12.4	1	Bal.	3.85	5.59	0.57	775, 800, 825, 850, 875, 900
E	11.5	2	Bal.	3.91	5.56	0.58	750, 775, 800, 825, 850, 875, 900

## Data Availability

Not applicable.
